# Reduced Thermal Conductivity in Nanostructured AgSbTe_2_ Thermoelectric Material, Obtained by Arc-Melting

**DOI:** 10.3390/nano12213910

**Published:** 2022-11-05

**Authors:** Javier Gainza, Federico Serrano-Sánchez, Oscar J. Dura, Norbert M. Nemes, Jose Luis Martínez, María Teresa Fernández-Díaz, José Antonio Alonso

**Affiliations:** 1 Instituto de Ciencia de Materiales de Madrid (ICMM), Consejo Superior de Investigaciones Científicas (CSIC), Sor Juana Inés de la Cruz 3, 28049 Madrid, Spain; 2 Departamento de Física Aplicada, Universidad de Castilla-La Mancha, 13071 Ciudad Real, Spain; 3 Departamento de Física de Materiales, Universidad Complutense de Madrid, 28040 Madrid, Spain; 4 Institut Laue Langevin, BP 156X, 38042 Grenoble, France

**Keywords:** thermoelectrics, neutron powder diffraction, layered nanostructuration, thermal conductivity

## Abstract

AgSbTe_2_ intermetallic compound is a promising thermoelectric material. It has also been described as necessary to obtain LAST and TAGS alloys, some of the best performing thermoelectrics of the last decades. Due to the random location of Ag and Sb atoms in the crystal structure, the electronic structure is highly influenced by the atomic ordering of these atoms and makes the accurate determination of the Ag/Sb occupancy of paramount importance. We report on the synthesis of polycrystalline AgSbTe_2_ by arc-melting, yielding nanostructured dense pellets. SEM images show a conspicuous layered nanostructuration, with a layer thickness of 25–30 nm. Neutron powder diffraction data show that AgSbTe_2_ crystalizes in the cubic *Pm-3m* space group, with a slight deficiency of Te, probably due to volatilization during the arc-melting process. The transport properties show some anomalies at ~600 K, which can be related to the onset temperature for atomic ordering. The average thermoelectric figure of merit remains around ~0.6 from ~550 up to ~680 K.

## 1. Introduction

The technological progress that humanity has witnessed in recent years has also led to an unstoppable increase in energy demand worldwide. In addition, society’s dependence on fossil fuels is a cause for concern, so renewable energy sources are becoming increasingly important. Thermoelectric materials, which can directly generate electricity from a temperature gradient, can be a key piece in the near future. These devices have several advantages, such as their reliability and absence of mobile parts as well as their environmental benignity. The efficiency of these materials is assessed by the thermoelectric figure of merit [1,2], *ZT*, a dimensionless parameter that is defined as *ZT* = (S^2^·*σ*/*κ*)·T, where S is the Seebeck coefficient, *σ* is the electrical conductivity, *κ* is the total thermal conductivity (which is the sum of the lattice and electronic contributions) and T is the absolute temperature.

Among the best thermoelectric materials, tellurides are perhaps the most studied compounds [3,4,5,6]. The most prominent, lead telluride (PbTe), has been widely used in the past century [7], and even today remains one of the best performing thermoelectrics [8,9,10,11]. Many approaches have been made in the past in order to enhance the thermoelectric performance of PbTe, such as the use of the band convergence concept [12] or deep defect level engineering [13], the implementation of solid solutions [14], the application of lattice strains [15], the introduction of different discordant atoms [8,16,17] and the design of all-scale hierarchical architectures [18]. One of the strategies that has been proven useful is the mixture of PbTe with AgSbTe_2_, resulting in a compound known as LAST-18 [11,19,20,21] with a remarkably good thermoelectric performance [11,22]. These kinds of chalcogenide compounds are the paradigmatic example of materials with a low thermal conductivity, due to the high bond anharmonicity [23,24], caused by the lone-pair electrons present in the structure [25].

Wernick and Benson synthesized AgSbTe_2_ for the first time in 1957 [26], and it has been well known in the thermoelectric community since then [22,27,28]. It was defined as a narrow band gap semiconductor with a rock-salt type structure where Ag and Sb atoms are located at random in the cationic sublattice [29]. The partial atomic ordering of these Ag/Sb atoms in the AgSbTe_2_ compound is an important feature when analyzing this material, since it strongly influences the electronic structure near the Fermi level [29]. Therefore, the precise determination of the Ag/Sb occupancy in this telluride has a significant relevance in terms of transport properties. This cation disordering is often found in other ternary cubic chalcogenide compounds, with the associated point defects usually leading to a poor reproducibility of the properties of these materials [30]. Recently, Roychowdhury et al. have proven that the atomic ordering can be achieved in AgSbTe_2_ with cadmium (Cd) doping [31]. This opens a new paradigm, since up until then, most thermoelectric materials were optimized by adding disorder [32].

The LAST compound AgPb_m_SbTe_2+m_ also exhibits the honor to be the bulk material in which nanostructuring was first reported [21,22,33,34,35]. Since then, this approach has been widely used in different thermoelectric compounds with the aim to improve the thermoelectric figure of merit, *ZT* [36,37,38,39,40,41]. Nanostructuring can produce, for instance, an increase in the Seebeck coefficient, by means of an increased quantization of the density of states [42], or a reduction in the lattice thermal conductivity, by means of an enhanced phonon scattering mechanism [43]. AgSbTe_2_ is reported to show a natural formation of nanoscale domains with different orderings on the cation sublattice [44], which is an example of increased phonon scattering, due to a nanostructuring effect.

We have previously used the arc-melting technique to synthesize different chalcogenides and nanostructured compounds, such as PbTe [45], GeTe [46,47,48], Bi_2_Te_3_ [49,50,51] or SnSe [39,52,53,54,55,56,57,58]. This synthesis method has the advantage to be very fast, compared to other techniques; the reaction itself happens in only a few seconds, and the entire process can be completed in several minutes. Furthermore, the sample is obtained in the form of dense ingots, which is a useful outcome when we think about the possibility to scale this process up. Here, we report on the synthesis and characterization of the ternary compound AgSbTe_2_ synthesized by arc-melting. Using this fast and straightforward technique, we can obtain highly dense pellets with nanostructuration in the layers, easily observed by scanning electron microscopy (SEM). The arc-melted compound has been studied by means of X-ray diffraction (XRD) and neutron powder diffraction (NPD), and we have measured its main thermoelectric properties; electrical resistivity, Seebeck coefficient and thermal conductivity, to gain some knowledge about its thermoelectric performance at high temperature.

## 2. Materials and Methods

AgSbTe_2_ was synthesized in an Edmund Buhler MAM-1 mini-arc furnace. The pressed pellet was placed in a water-cooled copper crucible, and was melted by a voltaic arc, created by a tungsten electrode in an inert Ar atmosphere. The melting process was repeated three times to ensure the homogeneity of the sample. The reagents were pure elements of Ag (99.9%, Goodfellow Metals, Cambridgeshire, UK), Sb (99.5%, Alfa Aesar, Haverhill, MA, USA) and Te (99.99%, Alfa Aesar, Haverhill, MA, USA), which were weighted (~1.5 g) and mixed, according to the stoichiometric ratio. A small part of the resulting ingot was cut and ground to powder to perform the structural characterization, and the rest of the sample was cold pressed in a Retsch (Haan, Germany) Pellet Press PP25 under an isostatic pressure of 10 MPa to do the transport measurements. This final pellet is typically ~10 mm in diameter and ~2 mm in thickness. The density of the cold-pressed pellet was ~90% of the theoretical crystallographic density. The high-temperature Seebeck coefficient was measured using an MMR technologies instrument under vacuum (10^3^ mbar) from room temperature up to ~750 K. Conventional van der Pauw geometry was employed to determine the electrical resistivity. The total thermal conductivity was calculated from the thermal diffusivity (α) using a Linseis LFA 1000 equipment, by the laser-flash technique. The thermal conductivity (κ) is determined from $\kappa =\alpha \cdot c_{p}\cdot d$, where *c_p_* is the specific heat, calculated using the Dulong–Petit equation, and *d* is the sample density.

Phase characterization was carried out for the pulverized sample using X-Ray diffraction (XRD) on a Bruker-AXS D8 (Karlsruhe, Germany) diffractometer run by DIFFRACTPLUS software (version 2.5.0, Bruker, Karlsruhe, Germany) in Bragg–Brentano reflection geometry with Cu Kα radiation (λ = 1.5418 Å). Furthermore, the NPD was used to characterize the crystal structure in detail. High-resolution patterns were collected in the D2B diffractometer at the Institut Laue-Langevin, Grenoble, France, in the high-flux configuration with a neutron wavelength λ = 1.549 Å, at 298 K. Around 2 g of the sample were measured in a vanadium can. The diffraction data were analyzed using the Rietveld method, employing the FULLPROF program (version Sept. 2018, Institut Laue-Langevin, Grenoble, France). The coherent scattering lengths of Ag, Sb and Te used in the refinement were 5.92, 5.57 and 5.80 fm, respectively. The profile parameters included in the refinement were the background as a set of refinable points, peak shape, asymmetry and FWHM parameters. The structural parameters included the scale factor, lattice parameters, atomic positions, isotropic atomic displacement parameters and occupancy factors. Scanning electron microscopy (SEM) images of an as-grown pellet were collected with a table-top Hitachi TM-1000 microscope (Hitachi, Japan).

## 3. Results & Discussion

### 3.1. Crystallographic Analysis by the XRD and NPD

**Figure 1:**
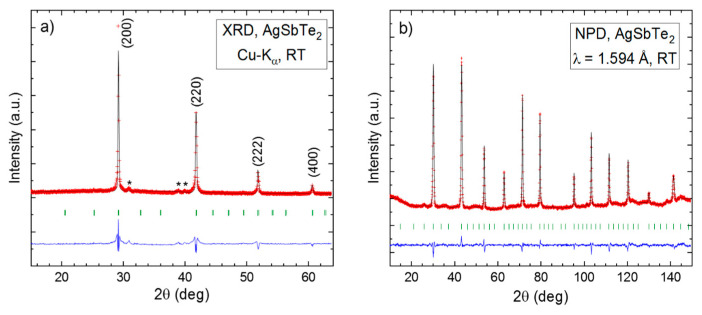
(**a**) XRD and (**b**) NPD patterns of AgSbTe_2_ at room temperature. The experimental points are shown in red, the calculated model in black and the difference in blue. The star denotes the known impurities detected in the X-ray pattern.

AgSbTe_2_ was obtained as a well-crystallized sample with negligible impurities (Figure 1). The laboratory XRD patterns display the expected AgSbTe_2_ cubic phase, defined in the space group *Pm*$\bar{3}$*m* with the lattice parameter a = 6.0788 Å. In Figure 1a, the peaks appear indexed in the mentioned cubic lattice. There are no additional reflections that could suggest a superstructure or a different space group. The pattern displays a slight preferred orientation effect and minor impurities of Ag_2_Te and Sb_2_Te_3_, as expected, according to previous literature reports [59].

A detailed structural investigation was performed by NPD at 295 K, which was essential as a bulk analysis to remove any orientation effects and to precisely determine the atomic displacement parameters (ADPs). The Rietveld refinement was performed using the CsCl-type structure, defined in the *Pm*$\bar{3}$*m* space group, which shows a good agreement with the observed pattern (Figure 1b). Previous reports have also proposed the F-centered *Fm*$\bar{3}$*m* space group to define the AgSbTe_2_ structure, but we could not improve the refinement of the primitive unit cell. Moreover, a structural description from the single-crystal diffraction data discarded a type-F lattice [60]. Thus, at *Pm*$\bar{3}$*m*, Ag and Sb atoms share randomly 3*c* (½, ½, 0) Wyckoff positions, Sb is additionally located at 1*b* (½, ½, ½) and Te at 1*a* (0, 0, 0) and 3*d* (0, ½, 0) Wyckoff positions. Table 1 lists the experimental set-up and Table 2, the determined structural parameters.

**Figure 2:**
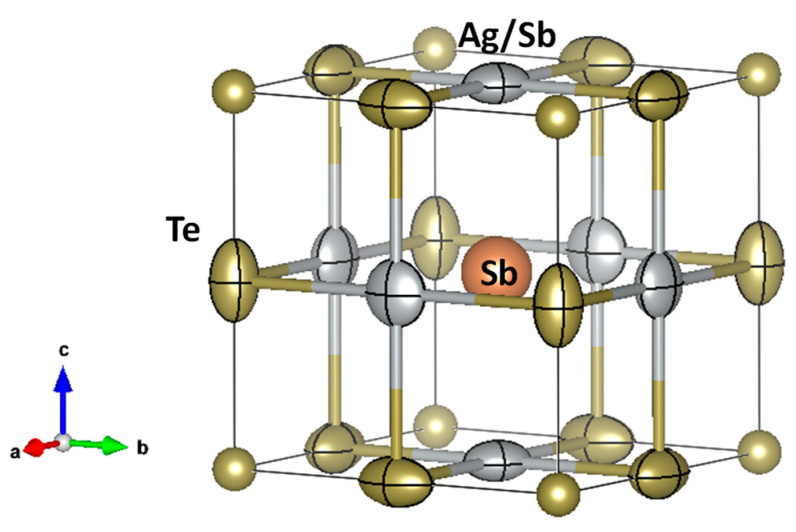
Crystal structure of AgSbTe_2,_ defined in the *Pm*$\bar{3}$*m* space group. The characteristic shape of the ellipsoids (elongated for Te1 and flattened for Ag/Sb2) are highlighted.

A slight Te deficiency at the 3*d* position is observed, which agrees with some Te volatilization during the arc-melting process, and these anion vacancies will increase the Fermi level and electron concentration. Figure 2 illustrates a view of the *Pm*$\bar{3}$*m* crystal structure, with the anisotropic atomic displacement ellipsoids (ADPs) for the 3*c* and 3*d* sites. This is a rock-salt-like structure, with Te and Sb as contiguous atoms and SbX_6_ (X = Ag, Sb) and TeTe_2_X_4_ octahedra. It is noteworthy that this model provides only one bonding distance, equivalent for every position. The highly anisotropic ADPs reflect the strong anharmonic bonding, which in turn has been ascribed to the lone s^2^ pair effect of Sb atoms [25]. The flat ellipsoids are perpendicular to the Sb-X bond and the elongated 3*d*-Te ellipsoids suggest more labile Te-Te, Ag/Sb-Te interactions and stronger Ag/Sb-Sb bonds, as well as repulsion effects with the origin at the 1*b*-Sb position. 

The crystal structure of AgSbTe_2_ is peculiar in the sense that Ag and Sb may be totally disordered, i.e., statistically distributed over the same crystallographic positions, as described in the cubic space group *Fm*$\bar{3}$*m*, or partially disordered, as described in the *Pm*$\bar{3}$*m* space group, and found in the present case, where part of Sb and Ag are still distributed at random over the 3*c* Wyckoff sites (see Table 2, Figure 2). Some authors [31] have found that, by means of Cd doping, the structure tends to increase the ordering; then Ag and Sb(Cd) become ordered by forming nanoregions. The fact that there are no thermal events in the DSC curves of AgSbTe_2_, as reported by Roychowdhury et al. [61], seems to suggest that AgSbTe_2_ remains in a partly ordered structure (*Pm*$\bar{3}$*m*), although the presence of partially ordered regions, at the nanoscale, that do not give rise to the superstructure reflections at intermediate temperatures, should not be discarded [29].

### 3.2. Scanning Electron Microscopy

**Figure 3:**
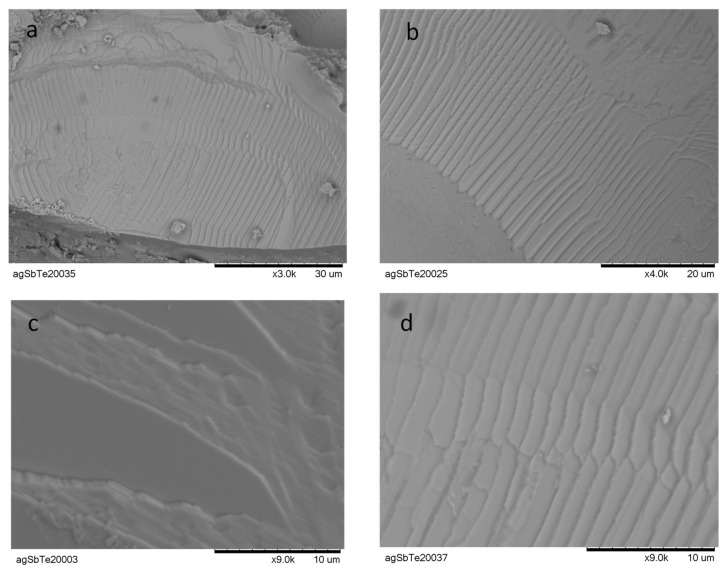
SEM micrographs with (**a**) 3000×, (**b**) 4000×, (**c**,**d**) 9000× magnification, illustrating the nanostructuration in the layers observed in this material, grown from arc melting.

The microstructure of the as-grown AgSbTe_2_ ingots has been investigated by high-resolution SEM, recorded in a table-top microscope. Some selected micrographs are shown in Figure 3. The material seems to be quite homogeneous, while it consists of a stacking of sheets, each of them presumably single-crystalline, with the large surfaces perpendicular to a crystallographic axis. This stacking of sheets seems to be a consequence of the arc-melting synthesis procedure, probably due to the inherent fast cooling protocol. Figure 3c,d assess a layer thickness in the 25–30 nm range. This strong nanostructuration in the layers, accounting for the ease of the cleavage of this material, is also responsible for the observed decrease of the thermal conductivity, as described below.

### 3.3. Thermoelectric Properties

**Figure 4:**
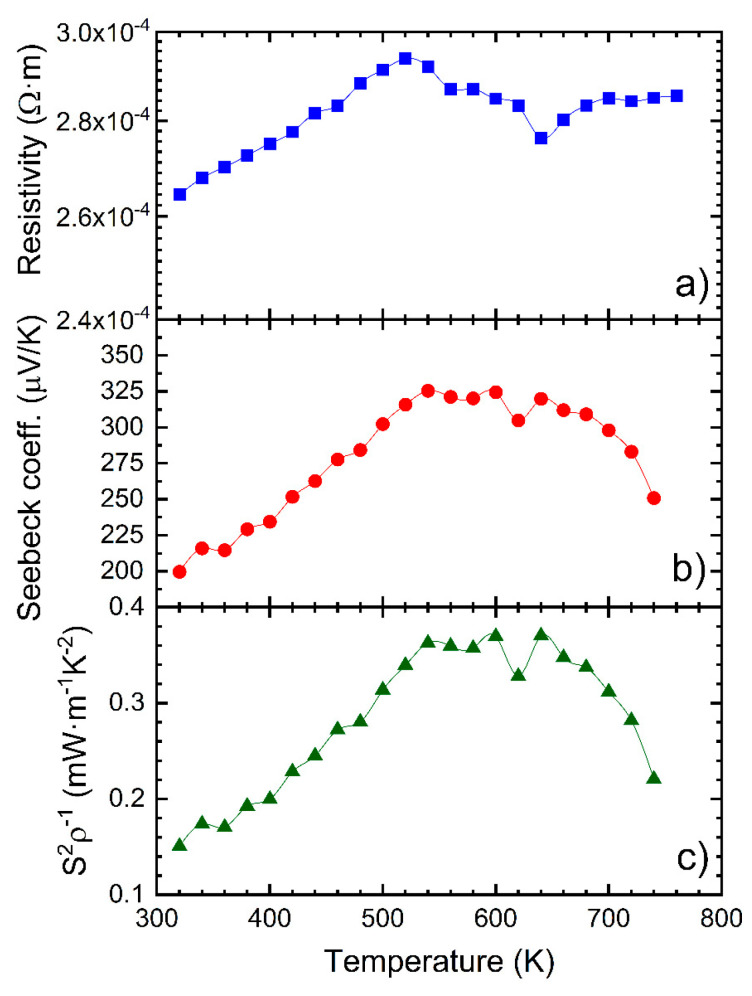
(**a**) Resistivity, (**b**) Seebeck coefficient and (**c**) power factor of the arc-melted AgSbTe_2_. The power factor is calculated from the experimental resistivity and the Seebeck coefficient.

Figure 4 displays the electrical transport properties of AgSbTe_2_. The resistivity (Figure 4a) shows almost constant values with the temperature, with a small variation from 2.6 × 10^−4^ to 2.8 × 10^−4^ Ω·m in the temperature range 300–770 K. It shows increasing values up to 550 K, with a small bump that has been related to the cation disorder at high temperatures [61], and which will also be apparent as a peak in the weighted, as we will discuss later. This is followed by a slight decrease in the resistivity, most likely a result of a minority carriers’ excitation. These values are above those that have been previously reported, within the range 0.5–2 × 10^−4^ Ω·m for pristine AgSbTe_2,_ prepared by melt-quench or mechanical alloying and SPS [62,63]. The Seebeck coefficient evolution with the temperature (Figure 4b) exhibits a steady increase from 200 µV K^−1^ to 340 V K^−1^ up to 540 K, a plateau up to 700 K and a stark reduction above 700 K, due to the bipolar contribution. This behavior is found in other AgSbTe_2_ samples, with a maximum value and temperature determined by the contribution of both carrier types in a semimetal as [64]:

$$S=\frac{S_{p}\sigma_{p}+S_{e}\sigma_{e}}{\sigma_{p}+\sigma_{e}}$$

AgSbTe_2_ displays a high Seebeck coefficient, due to its valley degeneracy and hole heavy effective band mass. Here, the samples display an increase of the Seebeck values above 300 µV·K^−1^, higher than those reported on the literature [62,63], possibly due to the lower values of the carrier concentration, which would also match the increased resistivity. This is opposite to the results obtained by the high-pressure high-temperature preparation [65]. 

The power factor (Figure 4c) follows the Seebeck coefficient evolution with maximum values close to 0.4 mW·m^−1^·K^−2^, in the range from 540 to 640 K [31,62,63]. These values are comparable to those reported in the literature (0.5–0.8), slightly limited by the higher electrical resistivity. AgSbTe_2_ presents a melting point slightly above ~800 K, and thus, the measurements have been performed up to ~760 K, to ensure the reproducibility of the results.

### 3.4. Thermal Conductivity

**Figure 5:**
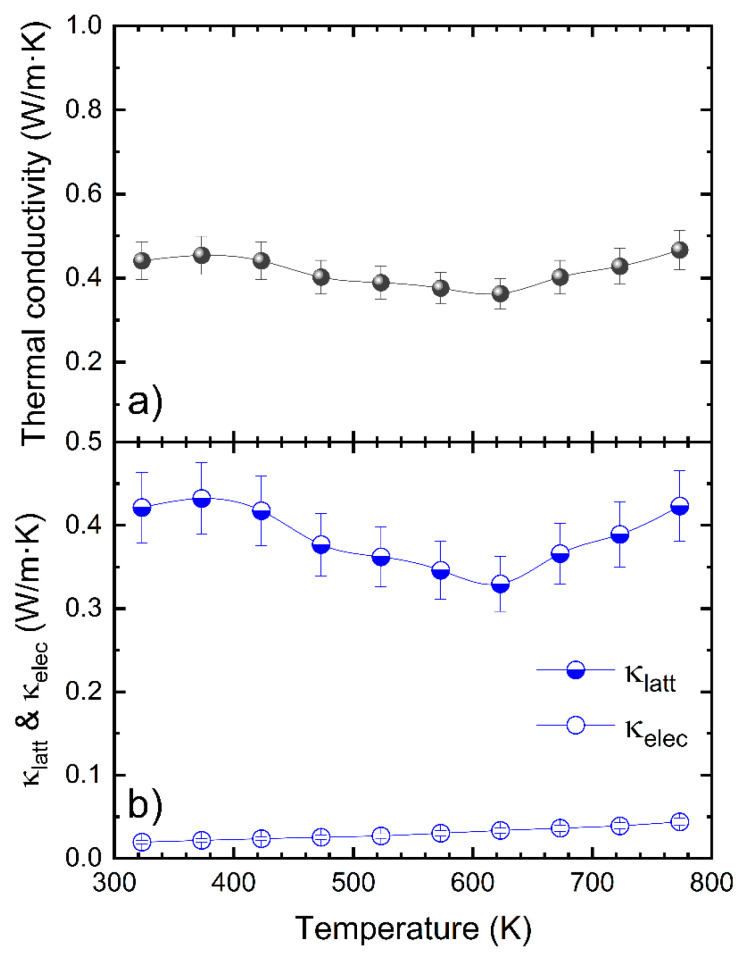
(**a**) Total and (**b**) lattice and electronic contributions to the thermal conductivity as a function of temperature for the AgSbTe_2_ compound. There is an increase in the lattice thermal conductivity at 623 K, probably related to the effect of the atomic ordering happening around that temperature range.

The total thermal conductivity vs. the temperature curve is shown in Figure 5a. It shows an almost constant value close to 0.4 W m^−1^ K^−1^, below the previously reported values for pristine AgSbTe_2_ [31]. The electronic and lattice contributions were determined by the Wiedemann–Franz law. The lattice thermal conductivity values are rather close to those of the total conductivity, due to a small electronic contribution (Figure 5b). AgSbTe_2_ exhibits an extremely low intrinsic thermal conductivity, which has been related to the anharmonic bonding, due to the lone pair effect [25]; it additionally presents a spontaneous nanostructuration, due to the different cationic ordering in the nanoscale [44,65,66]. Our specimen, prepared by arc melting, has even lower values than those reported in the literature, reaching down to 0.32 W·m^−1^ K^−1^ at 623 K, while previous reports show minimum values of 0.4 W·m^−1^ K^−1^. This enhanced phonon scattering, observed in our samples, is a consequence of the characteristic nanostructuration, obtained after arc-melting of the samples, as observed in the SEM images (Figure 3), and it was described in other thermoelectric materials [39,49,52].

The minimum value of the lattice thermal conductivity, observed at 623 K, followed by a conspicuous increase (Figure 5) can be explained, based on the effect of the atomic ordering happening at that temperature. It has been reported that this atomic ordering effect can be inferred from the unconventional temperature dependence of the transport properties [30], such as in the lattice thermal conductivity, as well as in the resistivity, the Seebeck coefficient and the weighted mobility.

**Figure 6:**
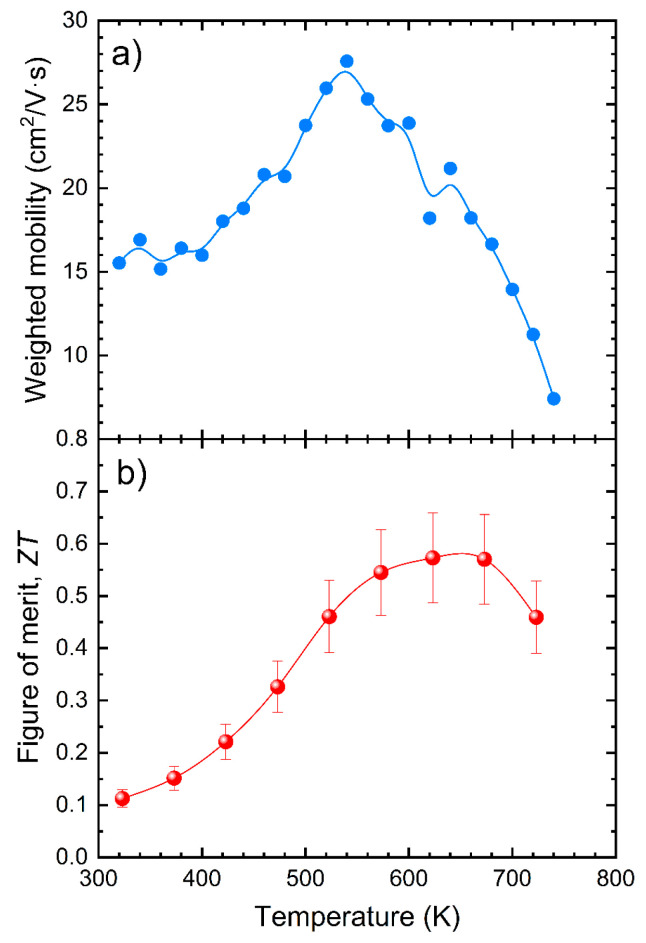
(**a**) Weighted mobility and (**b**) thermoelectric figure of the merit for the arc-melted AgSbTe_2_ compound.

In Figure 6a, the weighted mobility dependence on the temperature is shown. It increases up to 520–550 K, when the cationic disorder at a high temperature increases the carrier scattering. These values are much closer to those reported for the hole-mobility (~15 cm^2^ V^−1^ s^−1^), while much lower than those found for the electron mobility (~10^4^ cm^2^ V^−1^ s^−1^), as the heavy p-type band is the main contribution to the Seebeck coefficient. The maximum weighted mobility is consistent with the temperature evolution of the Seebeck coefficient and the resistivity, showing the significance of the cationic ordering in the electrical transport properties [31].

**Figure 7:**
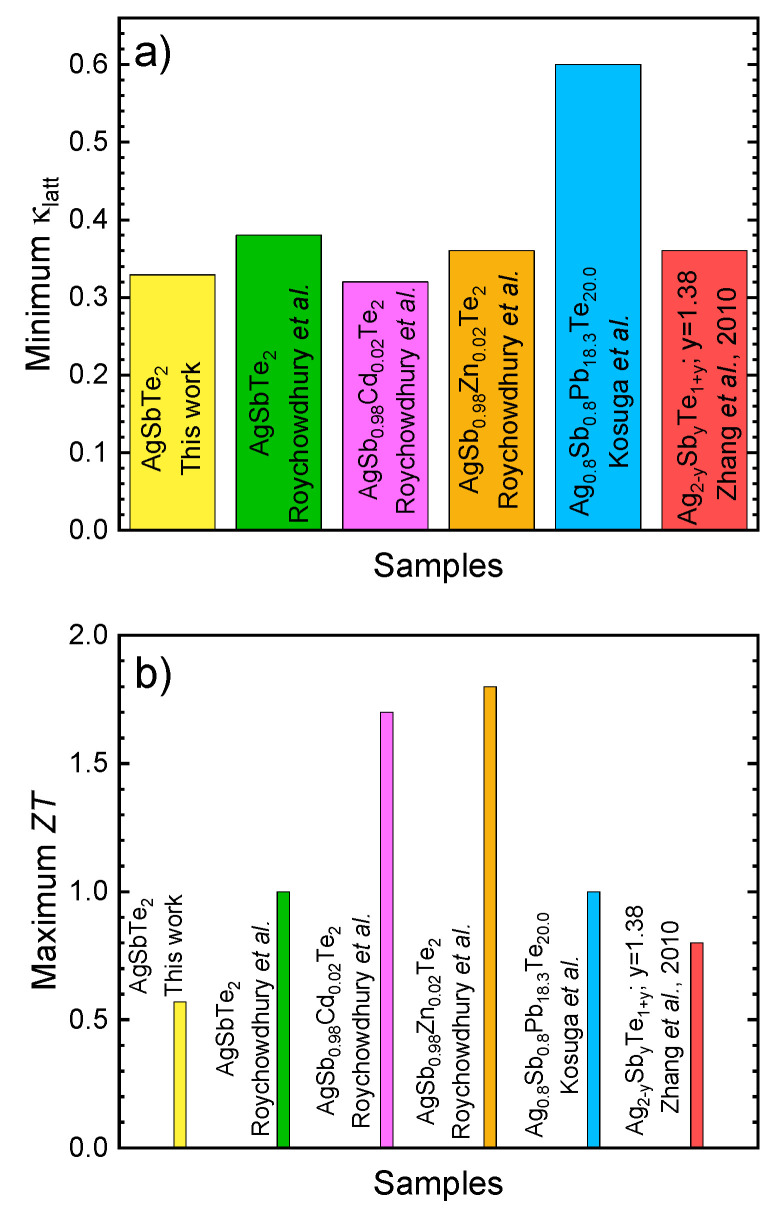
(**a**) Maximum thermoelectric figure of merit, *ZT*, and (**b**) minimum lattice thermal conductivity for several tellurides [31,61,67,68], compared with the composition analyzed in this work, AgSbTe_2_.

Overall, the figure of merit (Figure 6b) reaches a non-negligible value of 0.6 at 680 K, following the increase of the Seebeck coefficient, despite the reduced electrical conductivity. Nevertheless, these samples display the effect of an even more reduced thermal conductivity, as obtained by the nanostructuration of the arc-melted samples. The experimental data for this arc-melted compound are shown in Figure 7, together with other reported data for similar compositions, for the sake of comparison. Owing to this extremely low thermal conductivity, arc-melted samples are a suitable platform for the optimization of the electrical transport properties. Moreover, this synthesis procedure presents advantages over those previously described, since in a single step we obtain, by arc melting, a material in the form of an ingot, which can be directly implemented into a thermoelectric device, without requiring additional (and expensive) treatments, such as SPS, hot pressing, etc. 

## 4. Conclusions

We have prepared the telluride AgSbTe_2_ through the arc-melting technique, yielding a nanostructured dense pellet. The SEM images reveal a conspicuous layered nanostructuration, with layer thicknesses of 25–30 nm. The refinement of the crystal structure from the neutron powder diffraction data at RT has performed well, considering a *Pm*$\bar{3}$*m* space group and it reveals a tellurium deficiency that can be associated with the volatilization during the arc-melting process. This structure involves an intrinsic partial disordering of Ag/Sb, statistically distributed over the 3*c* Wyckoff positions. The possible atomic ordering reported for this compound can be inferred by an unconventional temperature dependence of the transport properties at high temperatures; this event can be detected at around ~600 K, when the weighted mobility and the lattice thermal conductivity, for example, show an anomaly in their behavior. The average thermoelectric figure of merit of this arc-melted compound remains at around ~0.6 from ~550 up to ~680 K, an important parameter to bear in mind to implement this material in practical devices.

## Figures and Tables

**Table 1:** NPD experimental parameters of AgSbTe_2_ at room temperature.

**Diffraction Parameters**	
Wavelength (Å)	1.594
2θ range (°)	0.07–159.97
2θ step (°)	0.05
Temperature (K)	295
Rietveld Refinement	3199 data points
No. of Parameters	67
**Structural parameters**	
Formula	AgSbTe_2_
Space Group	*Pm* $\bar{3}$ *m*
Z	2
a (Å)	6.0788(1)
V(Å^3^)	224.619(7)
Theoretical Density (g·cm^−3^)	7.168

**Table 2:** Structural parameters obtained from the refinement of the NPD data of AgSbTe_2_ at room temperature.

**Atomic Parameters**						
**Atom**	**Wyckoff site**	***x***	***y***	***z***	***U_eq_* (Å^2^)**	**Occ. (<1)**
Te1	3*d*	0.00000	0.50000	0.00000	0.027 (7)	0.82 (3)
Te2	1*a*	0.00000	0.00000	0.00000	0.014 (6)	1.0 (1)
Sb1	1*b*	0.50000	0.50000	0.50000	0.038 (8)	1.0 (1)
Ag	3*c*	0.50000	0.50000	0.00000	0.026 (5)	0.6667
Sb2	3*c*	0.50000	0.50000	0.00000	0.026 (5)	0.3333
**Atomic Displacement Parameters (Å^2^)**						
		***U^11^***	***U^22^***	***U^33^***	***U^12^***	***U^13^***
Te1		0.022 (5)	0.04 (1)	0.022 (5)	0.00000	0.00000
Sb		0.038 (8)	0.038 (8)	0.038 (8)	0.00000	0.00000
Ag		0.036 (5)	0.036 (5)	0.005 (6)	0.00000	0.00000
Sb2		0.036 (5)	0.036 (5)	0.005 (6)	0.00000	0.00000
**Agreement Factors**						**Bond Distance (Å)**
**R_I_(%)**		**R_p_(%)**	**R_wp_(%)**	**R_exp_(%)**	**χ^2^**	**d(Ag/Sb-Te)**
5.2		3.2	4.1	2.8	2.1	3.03939(6)

## Data Availability

Experimental raw data are available to the reader from lead contact upon reasonable request.
